# The development of 3-D, *in vitro*, endothelial culture models for the study of coronary artery disease

**DOI:** 10.1186/1475-925X-8-30

**Published:** 2009-10-28

**Authors:** Monica A Farcas, Leonie Rouleau, Richard Fraser, Richard L Leask

**Affiliations:** 1 Department of Chemical Engineering, McGill University, Montreal, Canada; 2 Department of Pathology, McGill University, Montreal, Canada

## Abstract

The response of the vascular endothelium to wall shear stress plays a central role in the development and progression of atherosclerosis. Current studies have investigated endothelial response using idealized *in vitro *flow chambers. Such cell culture models are unable to accurately replicate the complex *in vivo *wall shear stress patterns arising from anatomical geometries. To better understand this implication, we have created both simplified/tubular and anatomically realistic *in vitro *endothelial flow models of the human right coronary artery. A post-mortem vascular cast of the human left ventricular outflow tract was used to create geometrically accurate silicone elastomer models. Straight, tubular models were created using a custom made mold. Following the culture of human abdominal aortic endothelial cells within the inner lumen, cells were exposed to steady flow (Re = 233) for varying time periods. The resulting cell morphology was analyzed in terms of shape index and angle of orientation relative to the flow direction. In both models a progressive elongation and alignment of the endothelium in the flow direction was observed following 8, 12, and 24 hours. This change, however, was significantly less pronounced in the anatomical model (as observed from morphological variations indicative of localized flow features). Differences were also observed between the inner and outer walls at the disease-prone proximal region. Since morphological adaptation is a visual indication of endothelial shear stress activation, the use of anatomical models in endothelial genetic and biochemical studies may offer better insight into the disease process.

## Introduction

Cardiovascular disease is a leading cause of mortality and hospitalization in North America [1]. Atherosclerosis, a disease characterized by arterial wall fibrosis and lipid accumulation, occurs in elastic and large to medium size muscular arteries throughout the human vasculature, and is particularly evident in regions of curvature and bifurcation [2]. This focal predilection cannot be explained by lifestyle or genetic risk factors alone and has been linked to the response of endothelial cells (ECs) lining the luminal surface of blood vessels. It has been hypothesized that dysfunction of the endothelium leading to atherogenesis is stimulated by complex hemodynamic forces, such as wall shear stress [3].

Due to the difficulty of studying the endothelium *in vivo*, a number of *in vitro *systems have been developed with the aim of replicating the *in vivo *hemodynamics over a cultured monolayer of ECs in a controlled environment. These models include: parallel plate chambers [4-9]; cone and plate viscometer systems [10-14]; and three-dimensional, straight, tubular flow models [15-18]. Much has been discovered about the way in which endothelial cells respond to wall shear stress in such idealized models [19]. Most of these devices assume uniform shear stress and uniform cell response across the flow surface, allowing for easy quantification of average cell response.

Blood flow is characteristically three-dimensional, defined principally by the tortuous geometry of the vessel. The preservation of arterial geometry when studying wall shear stress has been shown to be of primary importance [20]. The complex wall shear stress patterns created *in vivo *are impossible to recreate in existing endothelial cell culture models, and their omission may be obscuring our understanding of shear induced EC dysfunction.

No model has accurately replicated the geometry of an artery for the creation of an anatomically accurate cell culture model. Such a model would better replicate the *in vivo *flow characteristics and shear stresses. The goal of our study was to create such a model of the human right coronary artery (RCA), and to culture ECs within it. An idealized straight tubular model was also developed for comparison.

Anatomical models, such as the one presented in this study, will be important in further studies of the activation of endothelial cell biochemical pathways involved in atherosclerosis, and in the testing of vascular devices and treatment strategies.

## Materials and methods

**Figure 1:**
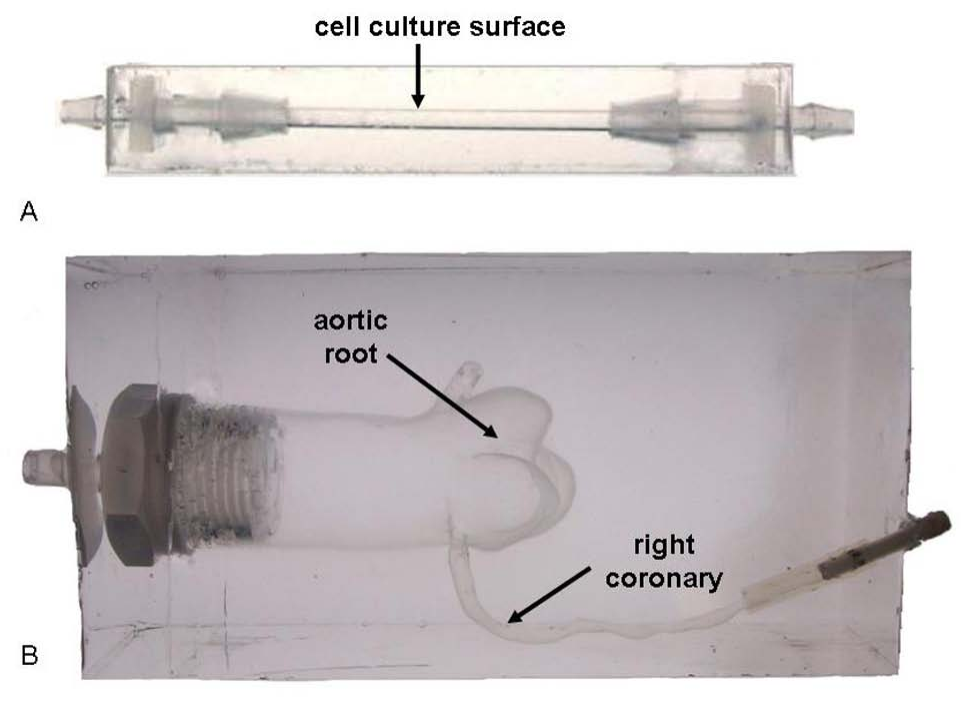
**(A) Simplified straight tubular model (B) anatomically accurate model of a 57 year male with no significant coronary artery disease who died of complications arising from colorectal surgery**.

Ethical approval was granted by McGill Institutional Review Board (A06-M62-04B) in accordance with Canada's tri-council policy on ethical conduct for research involving humans. For the preparation of geometrically accurate models, the entire left ventricular outflow tract (including the ascending aorta, the aortic root, and the coronary ostia as well as the inlet, proximal and acute marginal regions of the coronary arteries) of a mildly atherosclerotic post-mortem human heart was cast at physiologic pressure (100 mmHg) using Batson's No. 17 anatomical casting (Polysciences Inc., PA) [21]. The same procedure has been previously used to develop anatomically correct models for flow analysis [22]. The most successful cast was used to create Sylgard™ 184 silicone elastomer (Dow Corning, Canada) models for cell culture, using a low melting point alloy as an intermediate negative mold (Figure 1). Sylgard™ straight tubular models, 3.2 mm in diameter, were created using a custom made mold for comparison of cell response to steady one-dimensional laminar flow (Figure 1). The diameter of the straight tubular model was chosen to correspond to the average diameter of the inlet region of the anatomical model.

**Figure 2:**
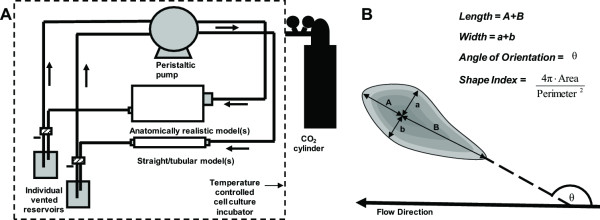
**(A) Schematic representation of the experimental flow system**. The closed-loop flow system used in this study consisted of the cell culture model(s), individual vented reservoirs, and a low-pulsatility 8-roller peristaltic pump (Ismatec, A-78002-34) coupled using biocompatible peroxide cured silicone tubing. All fittings and tubing were sterilized by autoclaving. (B) Illustration of the morphometric parameters calculated for each endothelial cell.

Preparation of the Sylgard™ models for cell culture was based on the procedure originally described by Qiu et al. [23]. The models were hydrophilized in 70% sulphuric acid, followed by sterilization, and coating with fibronectin (40 μg/ml) (F0895, Sigma-Aldrich, Canada). Human abdominal aortic endothelial cells (HAAECs) (HIAE-101, ATCC, VA), passage five, expanded within T-75 flasks, were cultured within the models using the following method. The cell suspension (5.0 ± 0.9 × 10^5 ^cells/mL) was pipetted into each model and allowed to settle for 2 minutes. The model was rotated and the process repeated until all sides were seeded. The model was then placed in the incubator strapped to a rotator (Labquake Rotor, Series 1104, Barnstead/Thermolyne) and slowly (8 rev/min) rotated for 4 hrs prior to flow experiments. The cell density (4.5 ± 0.8 × 10^4 ^cells/cm^2^) was verified using light microscopy. For the flow experiments, the models were then connected in parallel into a perfusion loop composed of individual vented media reservoirs and a low-pulsatility peristaltic pump (Ismatec A-78002-34, Canada), as seen in Figure 2A. The use of a peristaltic pump can introduce unwanted pressure fluctuations in the flow loop, however, these were minimal. The use of flow dampeners did not influence significantly cell shape in straight models (data not shown). The reservoir contained low-serum EC culture media (C-22210 PromoCell, Germany) containing 0.4% endothelial cell growth supplement, 0.1 ng/mL epidermal growth factor, 1 μg/mL hydrocortisone, 1 ng/mL basic fibroblast growth factor, 50 ng/mL amphotericin B, 50 μg/mL gentamicin (Promocell, C-39215), supplemented with 10% fetal bovine serum (Invitrogen, 26140-079), 1% penicillin streptomycin (Invitrogen, 15140-122) and supplemented with 6.7% dextran by weight (D4876, Sigma-Aldrich, Canada), to increase its viscosity to 3.5 cP and density to 1030 kg/m^3^. These values are in the physiological range for blood [24] and were used in order to match as closely as possible both a mean physiological Reynolds number of 233 [25] and an entrance wall shear stress of 20 dynes/cm^2 ^[26]. The entire apparatus was placed in a cell culture incubator, and cells were subjected to steady flow (119.5 ml/min, Re = 233) for varying time periods: 8, 12, and 24 hours. At the end of each experiment cells were fixed in 4% paraformaldehyde (P6148, Sigma-Aldrich, Canada) and stained with crystal violet (212525, BD Biosciences, Canada) for visualization under a light microscope.

**Figure 3:**
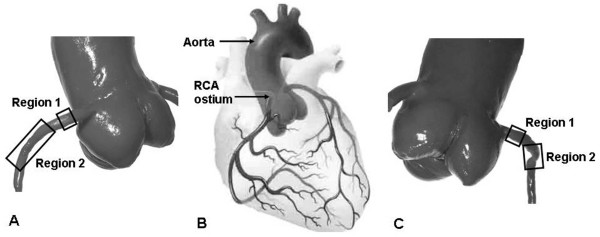
**The approximate location of the proximal RCA regions under study (Region 1 and Region 2) indicated on the anatomical cast; (A) ventral view (C) dorsal view**.

High resolution images captured at 100× total magnification were acquired using an inverted microscope and camera (DC300, Leica Microsystems, Canada) and processed for image analysis. The transparent nature of the models allowed us to directly visualize the cell surface. Due to the curvature of the model, only the center of the field of view could be used for analysis. Approximately 280-790 cells per experiment were analyzed in each location. For the anatomical model, two regions of the proximal RCA on both the inner (myocardial) and outer (pericardial) walls were analyzed. The first region (Region 1), approximately 5 mm in length, was selected in the relatively straight region near the ostium before the beginning of the first curvature (Figure 3). A second region (Region 2), approximately 7 mm in length, in the curved region of the proximal RCA was also analyzed.

To select cells within each image, an interactive Matlab™ 7.0 (Mathworks, CA) program was created. This software displays a filtered image and allows the user to select individual cells. Only cells in focus were taken for analysis. This type of semi-automated cell-picking is performed by allowing the user to manually select a pixel on the boundary of a deeply stained cell region. The algorithm then selects all pixels with intensities ≥ to the pixel chosen and subsequently selects all neighbouring pixels. The process is repeated until the whole cell is selected. Information about each particular cell (x, y of all the pixels) is stored, and the process is repeated for all discernable cells within each image.

A second algorithm processed the resulting cell information using Matlab™ image processing routines to compute the morphological parameters of each cell, including the angle of orientation (θ), the perimeter (P) and the area (A), as defined by Nerem et al. [27]. These are used to calculate the cell shape index (SI) as defined in Figure 2B. The SI characterizes the degree of cell elongation and is equal to 1 for a circle and 0 for a straight line. The angle of orientation is the angle between the cell major axis and the longitudinal axis of a section of the model. This was accomplished by positioning the model in the microscope stage so that the vessel walls were aligned along the vertical edges of the field of view at low magnification.

Shape index data was averaged and reported as means ± standard deviations. Statistical analysis was performed by parametric tests with the Graphpad statistical package (GraphPad, CA). Mean values were compared using a one-way analysis of variance (1-way ANOVA). If a significant difference was found among the means, multiple comparisons were performed using a Bonferroni post-processing test with a 95% confidence interval. A "p" value less than 0.05 was considered statistically significant. To compare two means a two-tailed parametric t-test was used. The distribution of the angle of orientation was tested for statistical significance using an F-ratio test on the variance of the sample.

## Results

HAAECs were successfully cultured in both models. Confluence was achieved in the three-dimensional models after 24 hours of static culture. Under static (no flow) conditions, the morphology of the cells in the three-dimensional models was similar to that seen in cell culture flasks.

**Figure 4:**
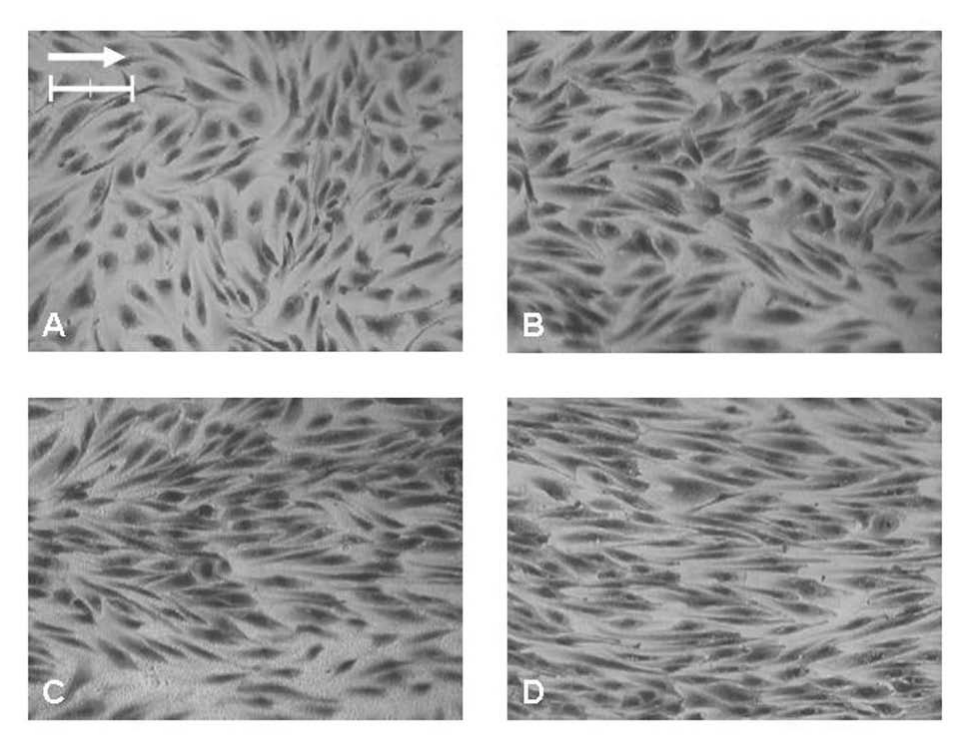
**Light microscope images of EC morphological changes in the tubular Sylgard™ model**. HAAECs were subjected to a steady laminar shear stress of magnitude 22 dynes/cm^2 ^for 8, 12, and 24 hours, (B-D) respectively. (A) Represents the no flow control. (Bar = 100 μm, Magnification = 100×). The arrow points in the direction of net flow.

**Figure 5:**
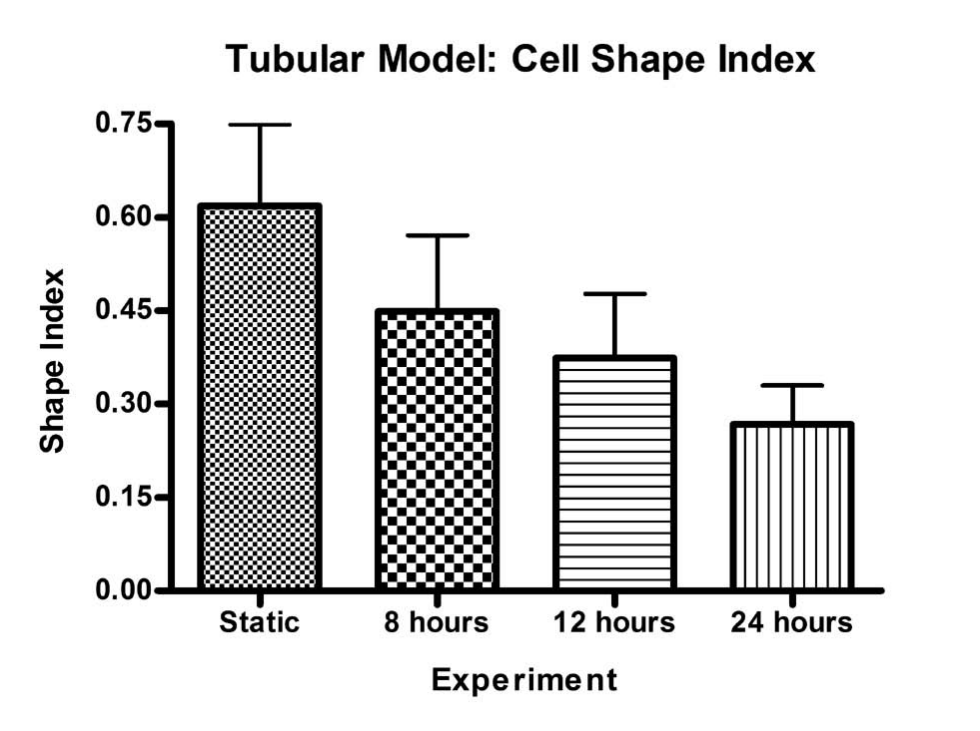
**Cell shape index time history for tubular model experiments, static, 8 hrs, 12 hrs and 24 hrs; SI = 1 corresponds to a perfect circle, while SI = 0 corresponds to a line; all values are expressed as mean ± standard deviation (n = 791, 562, 508, 276 respectively)**.

**Figure 6:**
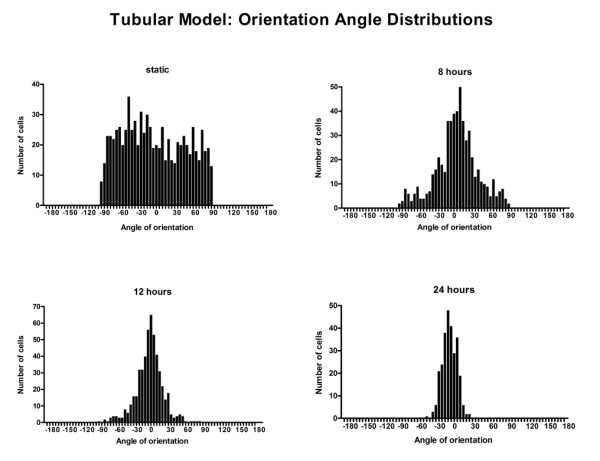
**Histograms illustrating the distribution of cell angles of orientation for the static (no flow) control, and the 8 hour, 12 hour, and 24 hour tubular model flow experiments (n = 791, 562, 508, 276 respectively)**.

Within the simplified tubular model, significant differences in the cell elongation (mean shape index) were found between the static and the 8, 12, and 24 hour perfusion experiments (p < 0.001 for all pair-wise comparisons, 1-way ANOVA, Bonferroni post-test) (Figure 4 and Figure 5). Under perfusion, progressive evidence of cell alignment in the axial flow direction was seen starting at 8 hours (Figure 4). This was followed by further cell elongation and alignment at 12 and 24 hours. The orientation (alignment angle) of the cells was normally distributed around 0°, the direction of flow (Figure 6). In the static control (no flow) a random distribution was observed. A significant narrowing (p < 0.05, F-test) of the distributions around 0° occurred as experimental flow time increased.

**Figure 7:**
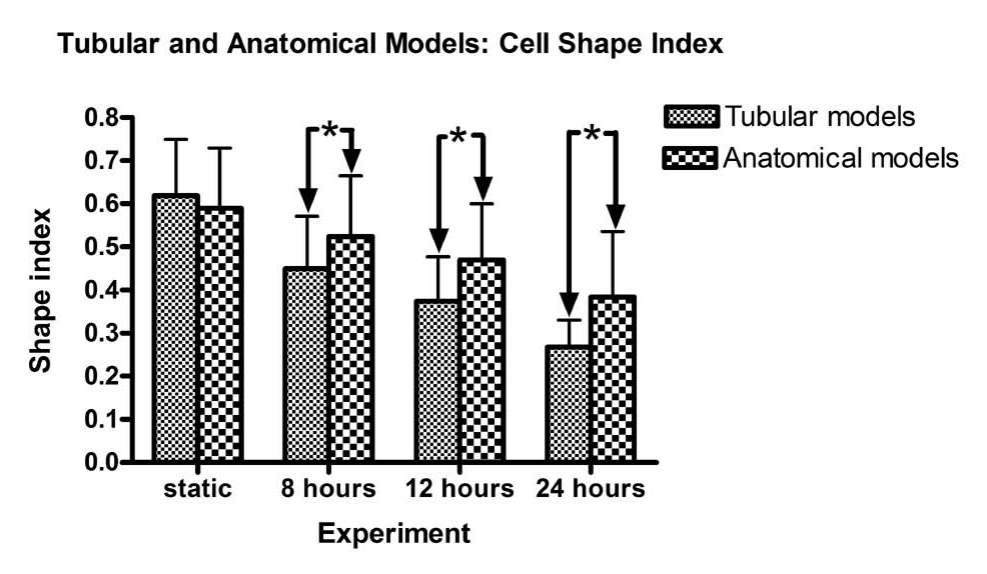
**Bar graph illustrating cell shape index time history for both the tubular and anatomical models**. The symbol (*) denotes a significant difference in the means; all values are expressed as mean ± standard deviation (n = 566, 562, 298, 344 respectively).

**Figure 8:**
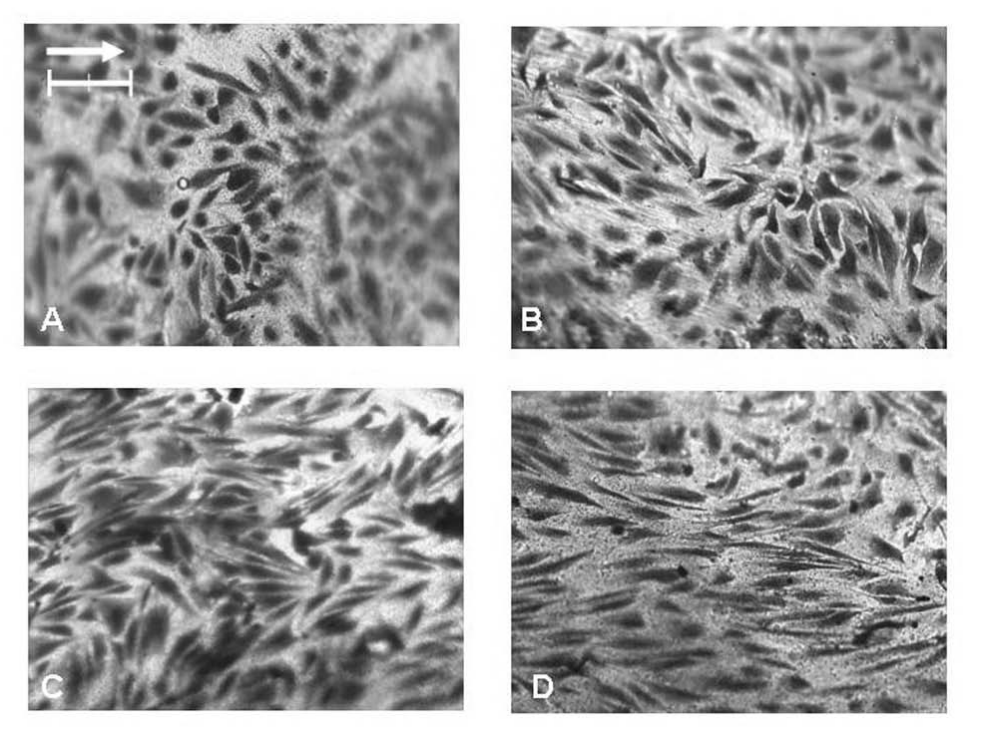
**Light microscope images of ECs in anatomical Sylgard™ models following 8, 12, and 24 hours, (B-D) respectively**. (A) represents the static (no flow) control. (Bar = 100 μm, Magnification = 100×). The arrow points in the direction of net flow. Certain images locations are blurred due to the local curvature.

The EC morphology in the relatively straight Region 1 was compared with the tubular model. There was no statistical difference between the mean shape indices of the static control models (anatomical vs. tubular, p > 0.05) (Figure 7). When subjected to flow, the pattern of HAAEC elongation and alignment with the vessel axis was less pronounced (Figure 8). In both regions, at all matched time points, the anatomical model displayed significantly less elongation than the tubular models (p < 0.01, ANOVA Bonferroni post-test). Interestingly, the mean cell shape in Region 1 of the anatomical model following 24 hours of flow was not statistically different from the 12 hour tubular model experiment (p > 0.05, ANOVA Bonferroni post-test). Similarly, the anatomical 12 hour experiment was also not different from the 8 hour simplified model experiment (p > 0.05, ANOVA Bonferroni post-test).

**Figure 9:**
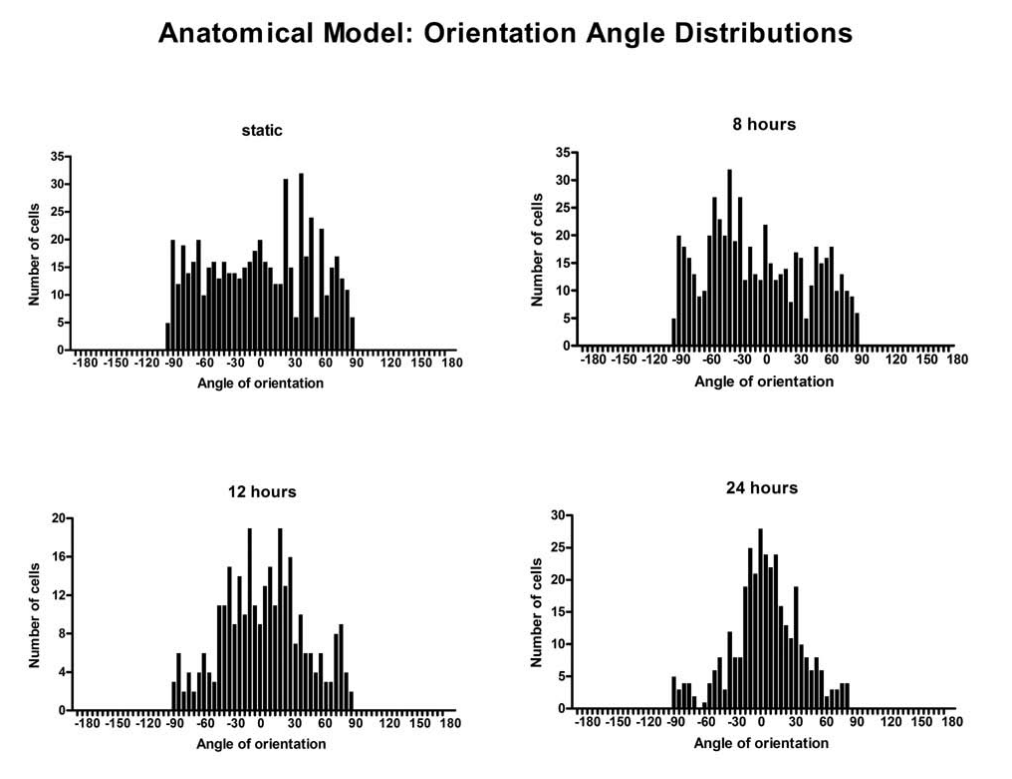
**Histograms illustrating the distribution of cell angles of orientation for the static, 8 hour, 12 hour, and 24 hour anatomical model flow experiments (n = 566, 562, 298, 344 respectively)**.

Region 1 alignment angle histograms for anatomical model experiments were observed to be more widely distributed around 0° (Figure 9). In general, there was a significant decrease in the angle variability with flow; however, for the anatomical model this was true only following 8 hours of flow. At all time points, significantly less alignment was seen in the anatomical model.

**Figure 10:**
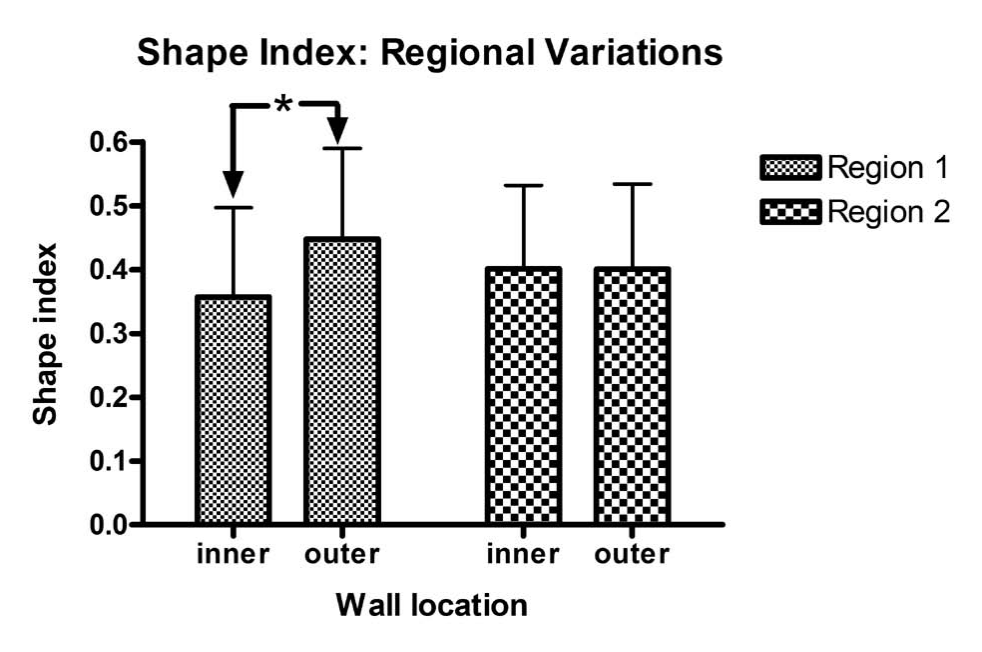
**Bar graph illustrating cell shape index variation between the inner and outer walls of the anatomical model in Region 1 and 2**. The symbol (*) denotes a significant difference in the means (n = 325, 266, 112, 86 respectively).

**Figure 11:**
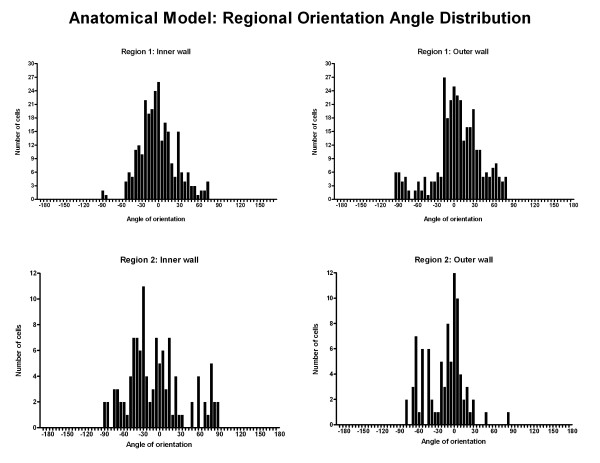
**Histograms illustrating the distribution of cell angles of orientation for the inner and outer walls of the anatomical model in Region 1 and 2 at the 24 hour time point (n = 325, 266, 112, 86 respectively)**.

Region 1 and 2 of the proximal RCA in the anatomical model were further analysed to investigate possible differences in cell morphology between the inner (myocardial) wall and the outer (pericardial) wall at the 24 hour time point. A significant difference in shape index was found in the relatively straight section at the inlet region near the ostium (Region 1), with cells on the inner wall displaying a more elongated morphology (p < 0.0001, ANOVA Bonferroni post-test) (Figure 10). In contrast, in the proximal region (Region 2), no significant difference was observed between the inner and outer wall cell shape index. This trend was also observed in the orientation angle, Figure 11. A statistically significant difference between the variance of the angle of orientation between the two walls was observed (P < 0.0001, F-test) in the inlet region (Region 1), whereas this was not the case in the proximal region (Region 2).

## Discussion

In this study, we developed an anatomical three-dimensional flow model of a human right coronary artery which was able to support cultured human abdominal aortic endothelial cells. We have shown that these cells remain adherent under a physiologic magnitude of wall shear stress during steady flow and alter their morphology in response to flow differently in uniform cell culture flow chambers (Figures 4 and 8). To our knowledge, the anatomical model developed in this study is the first endothelial cell culture model with realistic arterial geometry.

Flow studies have shown that the preservation of arterial geometry is fundamental in reproducing *in vivo *wall shear stress patterns [28,29]. Even the most accurate flow models of human coronary blood flow have been incapable of capturing the flow through the ostium [30,31]. Our model accurately replicates the geometry driven flow through the ostium and the proximal RCA. The morphological adaptation of endothelial cells to wall shear stress in this region was analyzed because the proximal region of human coronary arteries is a high risk area for eccentric intimal thickening and atherosclerosis [32,33].

EC response to shear has been widely documented in simplified *in vitro *models [14,34-43]. Studies involving such models have shown that low [44], high [45] and oscillatory [46] wall shear stress can cause cultured ECs to respond differentially by changing their structure and function [47-50]. Much of this work has been conducted in parallel flow chambers because of their relative simplicity and commercial availability. These devices assume uniform shear stress and uniform cell response across the flow surface, allowing for easy quantification of average cell response. Unfortunately, such simplified models fail to mimic the diversity of *in vivo *arterial wall shear stress patterns and the resulting biomechanical environment. By exposing all cells to the same level of shear, these studies mask local *in vivo *cell-cell signalling interactions [51] and therefore may not be representative of the endothelium in general.

Spatial gradients in wall shear stress have been shown to create a heterogeneous cell response in culture. Particularly, modified parallel plate chambers have been used to generate shear gradients present in flow separation and reattachment. In these studies a rectangular barrier is placed perpendicular to the flow direction to try to create three defined areas of disturbed flow (reversal, reattachment, and recovery). Morphological and functional differences have been identified in the three regions [52,53]. However, the physiological significance of these changes is difficult to interpret, since this is an unnatural geometry. More recent work concerning more realistic gradients was performed by LaMack and Friedman [54]. Despite such limitations, these studies have provided support for the endothelial heterogeneity hypothesis of focal atherosclerosis [55].

The model we have developed will allow us to directly determine the response of endothelial cells to realistic spatial and temporal gradients in wall shear stress present in human coronary arteries. The realistic spatial flow pattern produced in our model causes significant regional differences in cell morphology in the disease-prone proximal region (Figure 10). When compared to a straight tubular model, we observed significantly less cell elongation and cell alignment in the flow direction in the anatomical models at all time points (Figure 8). These results suggest that the dramatic endothelial elongation seen in simplified models may not be representative of true EC behaviour to realistic spatial wall shear stress patterns. Local heterogeneity in endothelial cell response was evident in Region 1, where a significant difference between the outer and inner wall morphology was demonstrated. Moreover, there is little consistency in endothelial cell morphology in the proximal region, suggesting possible phenotypical differences. This preliminary work has highlighted the local response of ECs to shear stress and is the first step in evaluating the role of spatial gradients in wall shear stress created by a realistic geometry in the development of focal atherosclerosis. We believe it is important to preserve the *in vivo *anatomy to properly investigate the mechanism of shear induced endothelial cell dysfunction in atherosclerosis, since geometrical variation has implications in cell to cell signalling, upstream release of cytokines and blood component/EC interaction.

Few three dimensional *in vitro *and *in vivo *morphological studies are available to compare our results with others. The shape index values obtained for the straight/tubular models are lower than those reported by Helmlinger et al. [56] for bovine aortic endothelial cells exposed to 25 dynes/cm^2 ^in a parallel plate chamber, namely 0.65 at 8 hours and 0.45 at 24 hours. This may be due to differences in cell type or differences in the hemodynamic stimuli created by a tubular model. The area targeted for morphometric analysis (the nuclear region, rather than the cell boundary) and staining technique may also contribute to this difference. Our simplified model is in closer agreement with the findings of Nerem et al. [57] who analyzed cell shape directly from silver stained arterial tissue and observed shape index of 0.35 ± 0.02. Ziegler et al. [58] employed similar Sylgard™ tubular models with a greater diameter of 6 mm and analysed endothelial cell shape. However, in this study, the cell monolayer was only exposed to very low shear stress, with a maximum shear stress of 6 dynes/cm^2^, resulting in a reported shape index similar to our static models.

Protein and gene expression studies performed in simplified *in vitro *geometries have greatly contributed to the understanding of atherosclerotic development *in vivo*. Our work provides evidence that a late step (morphological adaptation) in the cascade of events that occurs after the onset of flow is influenced by the presence of realistic spatial wall shear stress gradients. Once, validation of the flow patterns have been fully performed in these anatomically realistic models, analysis of the cell phenotype can be performed by extraction of cells (proteins and RNA) from the models [59]. Due to the transparent nature of the model, local observations on the expression through confocal imaging are also possible.

A number of assumptions were made for the development of our model that are similar to existing cell culture models. We have used a monolayer of ECs to approximate the arterial wall, ignoring interactions with other cells of the artery wall. We have also used cell culture media as a blood substitute, and have not considered the effects of blood components. Other mechanical forces, such as transmural pressure and cyclic strain, are known to affect endothelial cells. Although the model we have created is made from a material with distensible properties, we have not attempted to quantify or replicate the cyclic strain associated with pressure fluctuations or myocardial contraction. It is important to note that endothelial cell response to pulsatile flow can be significantly different [60-64]. These effects have not been accounted for in the current study. Indeed, in this model, steady flow was assumed.

It is also important to remember that there are significant variations in the anatomical structure of human coronary arteries[65]. Therefore, it is difficult to generalize our patient-specific findings to all the cases that may be encountered in the human coronary arterial tree. To be able to draw such conclusions, the current study needs to be repeated with several other casts.

In the right coronary artery, it has been estimated that over 60% of flow occurs during diastole, and this can rise to 80% in the presence of aortic valve disease [25]. Hence, as a first approximation, we have assumed steady retrograde flow in the ascending aorta to capture diastolic coronary flow. We have not considered the time varying characteristics of blood flow, geometrical changes due to the motion of the RCA during the cardiac cycle, or the effect of branches. Branching patterns, particularly in the RCA, can vary significantly between individuals. However, compared to the left coronary artery, branches in RCA tend to be small relative to main trunk of the artery.

## Conclusion

In this study we have presented the groundwork for a new anatomically realistic *in vitro *cell culture model which can be used to better simulate the complex *in vivo *wall shear stress patterns present in the human RCA. This model showed significant differences in EC morphology, even when compared to the most advanced idealized flow systems. Since structure and function are intimately linked, it is concluded that realistic wall shear stress patterns created by anatomic geometries are vital to the study of shear induced atherosclerosis. Our model will be beneficial not only in further elucidating the role of ECs in atherosclerosis, but also in the design and testing of vascular devices and treatment strategies.

## Competing interests

The authors declare that they have no competing interests.

## Authors' contributions

MAF designed and performed the research, collected, analyzed and interpreted the data, and performed statistical analysis. LR carried out preliminary experiments, gathered and analyzed part of the morphology data, and participated in the design of the study. RF provided access to human tissue. RLL funded the research and contributed to the troubleshooting and the experimental design. All authors read and approved the final manuscript.
